# Representation of Cone-Opponent Color Space in Macaque Early Visual Cortices

**DOI:** 10.3389/fnins.2022.891247

**Published:** 2022-06-20

**Authors:** Xiao Du, Xinrui Jiang, Ichiro Kuriki, Toru Takahata, Tao Zhou, Anna Wang Roe, Hisashi Tanigawa

**Affiliations:** ^1^Department of Neurosurgery of the Second Affiliated Hospital, Interdisciplinary Institute of Neuroscience and Technology, School of Medicine, Zhejiang University, Hangzhou, China; ^2^MOE Frontier Science Center for Brain Science and Brain-Machine Integration, School of Brain Science and Brain Medicine, Zhejiang University, Hangzhou, China; ^3^Key Laboratory of Biomedical Engineering of Ministry of Education, College of Biomedical Engineering and Instrument Science, Zhejiang University, Hangzhou, China; ^4^Department of Information and Computer Sciences, Graduate School of Science and Engineering, Saitama University, Saitama, Japan

**Keywords:** intrinsic signal optical imaging, DKL color space, V1, V2, V4, functional domain, visual cortex

## Abstract

In primate vision, the encoding of color perception arises from three types of retinal cone cells (L, M, and S cones). The inputs from these cones are linearly integrated into two cone-opponent channels (cardinal axes) before the lateral geniculate nucleus. In subsequent visual cortical stages, color-preferring neurons cluster into functional domains within “blobs” in V1, “thin/color stripes” in V2, and “color bands” in V4. Here, we hypothesize that, with increasing cortical hierarchy, the functional organization of hue representation becomes more balanced and less dependent on cone opponency. To address this question, we used intrinsic signal optical imaging in macaque V1, V2, and V4 cortices to examine the domain-based representation of specific hues (here referred to as “hue domains”) in cone-opponent color space (4 cardinal and 4 intermediate hues). Interestingly, we found that in V1, the relative size of S-cone hue preference domain was significantly smaller than that for other hues. This notable difference was less prominent in V2, and, in V4 was virtually absent, resulting in a more balanced representation of hues. In V2, hue clusters contained sequences of shifting preference, while in V4 the organization of hue clusters was more complex. Pattern classification analysis of these hue maps showed that accuracy of hue classification improved from V1 to V2 to V4. These results suggest that hue representation by domains in the early cortical hierarchy reflects a transformation away from cone-opponency and toward a full-coverage representation of hue.

## Introduction

Color information of light input to the eye is initially expressed as differences in the responses of three types of cone photoreceptors (hereafter referred to as L, M, and S cones), which have peaks of sensitivity at long, medium, and short wavelengths, respectively, in the retina. Retinal ganglion cells compare the output of these cones and send signals to neurons in the lateral geniculate nucleus (LGN). The LGN has a layered structure that contains three classes of neurons that differ in their color sensitivity based on cone opponency (De Valois et al., 2000). Most of the neurons in the magnocellular layers are sensitive to changes in luminance, which is composed of the sum of L and M cone signals, and has no selectivity for color. Neurons in the parvocellular layers are tuned to luminance and primarily tuned to the difference between L- and M- cone signals (“L–M”), while neurons in the koniocellular layers are tuned to the difference between S cone signals and the sum of L- and M- cone signals [“S–(L+M)”] (Hendry and Yoshioka, 1994). The direction of color change to which these three classes of LGN neurons are sensitive corresponds well with the psychophysically defined “cardinal directions” of color space (MacLeod and Boynton, 1979; Krauskopf et al., 1982; Derrington et al., 1984). In the primary visual cortex V1, which receives signals from the LGN, the majority of color selective neurons are tuned not just to the cardinal direction but also to an intermediate color direction (Lennie et al., 1990; Hanazawa et al., 2000; Wachtler et al., 2003). This indicates that the cone-opponent signals in the LGN are integrated into V1 to generate a broader range of chromatic tuning properties.

Several studies indicate that the generation of hue maps is initiated in V1 and further transformed in V2 and V4. Intrinsic signal optical imaging (ISOI) studies have revealed spatial arrangements of hue preferences (hue maps) in macaque V1 (Xiao et al., 2007), V2 (Xiao et al., 2003), and V4 (Tanigawa et al., 2010; Li et al., 2014), where different colors activate overlapping but spatially distinct cortical areas (domains, 0.2∼0.8 mm in width). Neuronal clusters with similar color preferences corresponding to these domains have been identified using two-photon calcium imaging (Garg et al., 2019; Liu et al., 2020; Chatterjee et al., 2021). In V1, there is a bias of neural responses to endspectral colors (such as red and blue; Tootell et al., 1988; Yoshioka and Dow, 1996; Xiao et al., 2007; Salzmann et al., 2012; Garg et al., 2019); this bias is mitigated in V2 and virtually absent in V4 as the response to different hues becomes more balanced (Liu et al., 2020). However, the bias to endspectral hues in V1 could have resulted from unequal stimulus strength in cone contrast. Using two-photon calcium imaging and colors based on cone opponency, clusters of V1 neurons preferring particular colors were identified (Chatterjee et al., 2021). However, no study has compared the representation of color by domains in V1, V2, and V4 based on cone opponency, making it difficult to evaluate how each stage progresses from cone opponent origins.

In this study, analogous to functional maps of contour orientation domains in the visual cortex, using ISOI and decoding methods, we compared the representation of hues in the cone-opponent color space by functional domains that constitute the cortical hue map in macaque V1, V2, and V4. These comparisons revealed differences among V1, V2, and V4 in the size and distribution of hue-selective domains, and the amount of information available to discriminate hue by activity patterns. These findings support a transformation in the cortical hue representation by domains from a cone-opponency-dependent to a more balanced and distinctive hue representation.

## Materials and Methods

### Animal Preparation

Four hemispheres (cases 1–4) from two male (monkeys D and G) and one female (monkey Y) adult rhesus monkeys (*Macaca mulatta*, 4–7 kg) were used for experiments. Case 1 was obtained from the right hemisphere of monkey D, cases 2 and 3 were obtained from the right and left hemispheres, respectively, of monkey G, and case 4 was obtained from the left hemisphere of monkey Y. All procedures were approved by the Institutional Animal Care and Use Committee of Zhejiang University and conformed to the guidelines of the National Institute of Health Guide for the Care and Use of Laboratory Animals. Animals were sedated with Zoletil (2.5 mg/kg, i.m.), intubated with an endotracheal tube, and placed in a stereotaxic apparatus. They were artificially ventilated, and anesthesia was maintained with isoflurane (1.5–2%) during the surgical procedure. The heart rate, arterial oxygen saturation, end-tidal CO_2_ were continuously monitored, and rectal temperature was maintained at 37°C throughout the experiment. Before the first imaging session, under sterile surgical conditions, craniotomy and durotomy were performed to expose visual areas V1, V2, and V4. Native dura was replaced with a transparent artificial dura (Tecoflex, Thermedics Polymer Products). A PEEK imaging chamber was implanted over the exposed cortex and sealed with a PEEK cap. The animals’ eyes were dilated with 1% atropine sulfate eye drops and fitted with contact lenses of appropriate curvature (Danker Laboratories Inc., Sarasota, FL, United States) to focus on a computer screen. The positions of the foveae were back-projected onto the screen using a reversing ophthalmoscope at regular intervals throughout the imaging. During the imaging session, the anesthesia was maintained with propofol (5 mg/kg/h, i.v.) and the paralysis with vecuronium bromide (0.1 mg/kg/h, i.v.).

### Visual Stimulation

Visual stimuli were generated using a stimulus generator (ViSaGe MKII; Cambridge Research Systems, United Kingdom) and presented on an LCD monitor (272G5DJEB, Philips, Netherlands) with a resolution of 1,920 × 1,080 pixels and a 60 Hz refresh rate. The monitor was positioned 57 cm in front of the monkey’s eyes, creating a field of view of 59.7° × 33.6° visual angle. The monitor was calibrated by SpectroCAL MKII Spectroradiometer (Cambridge Research Systems) to achieve a linear gamma. Electromechanical shutters (VCM-D1, Vincent Associates, NY, United States) were placed in front of the monkey’s eyes. Each of the shutters was opened in pseudo-random order for stimulus presentation to each eye. During the stimulus intervals, the shutters were closed. Visual stimulation and eye shutters were controlled by custom software written in MATLAB (The MathWorks, Inc., MA, United States).

To examine the distribution of preference for hues in the cone-opponent color space in the visual cortices, we presented a visual stimulus of 10% luminance-contrast square-wave gratings covering the entire screen (0° or 90° orientation, 1–2 cycle/degree, 2–4 cycle/s). Drifting direction was randomized on every presentation. The dark stripes of the gratings always had a gray color (*x* = 0.33, *y* = 0.33 in the CIE 1931 chromaticity; 65.5 cd/m^2^). The bright stripes of the gratings had one of eight different colors or a gray color at the same luminance (80 cd/m^2^). The hues of gratings were defined within the isoluminant plane of the DKL color space (MacLeod and Boynton, 1979; Krauskopf et al., 1982; Derrington et al., 1984) based on the Smith and Pokorny cone fundamentals (Smith and Pokorny, 1975). Within the isoluminant plane, the excitation of the L and M cones covaried along one axis (L – M axis), keeping their sum constant, while the excitation of the S cones was constant. Conversely, along the second axis (S axis), only the excitation of the S cones changed, whereas the excitation of the L and M cones remained unchanged. Hues were defined by their chromatic direction given by the polar angle (hue angle) ranging from 0 to 360 degree from an arbitrarily defined gray point, metamer of equal-energy white (*x* = 0.33, *y* = 0.33). The stimulus saturation was normalized to the maximum extent of color available on the LCD screen. They were 7 and 70% of increment for the L- and S-cones, respectively, in terms of the cone contrast with respect to those for the background gray. The hue saturation, defined as the distance between each hue and the gray point, was set equal in the DKL color space. The entire locus of the hue fits in the gamut of the LCD screen. The CIE 1931-xy chromaticity coordinates of the eight hues and gray color are shown in Supplementary Table 1. All stimulus conditions and one blank (no stimulus) condition were presented in a pseudo-random order and were repeated at 40 times in each stimulus. After a 1-s pre-stimulus period, a visual stimulus was presented for a 3.5 s (stimulus period), followed by an interval of at least 8 s before the next pre-stimulus period. During the pre-stimulus periods, stimulus period in the blank condition, and interval, the monitor was filled with a gray background (*x* = 0.33, *y* = 0.33; 72.7 cd/m^2^).

To examine the distribution of preference for color and orientation in the visual cortices, as revealed in previous studies (Lu and Roe, 2008; Tanigawa et al., 2010), we presented a circular patch filled with isoluminant red/green or luminance-contrast (100%) black/white drifting sinusoidal gratings (0° or 90° orientation, 1–2 cycle/degree, 2–4 cycle/s). The gratings had an average luminance of 40 cd m^–2^, identical to the background luminance (*x* = 0.319, *y* = 0.318). The CIE 1931-xy chromaticity coordinates of the red, green, and white are also shown in Supplementary Table 1.

### Optical Imaging and Data Analysis

Detailed imaging and data analysis methods have been described previously (Tanigawa et al., 2010, 2016; Hu et al., 2020). Prior to the imaging, the chamber cap was opened under sterile conditions, and the exposed cortex was stabilized with warm 3% agarose and a glass disk. Under 630 nm illumination, images of light reflectance from a portion of the exposed cortex containing V1 and V2 or V2 and V4 were captured using a CCD camera (1,308 × 1,080 pixels, MV1-D1312(IE)-160-CL, Photonfocus, Switzerland) with a tandem lens system focused on the cortical surface (10.5 mm × 8.7 mm). Images were digitized using Imager 3001 (12-bit resolution, Optical Imaging Ltd, Israel) at 4 frames/s for 4.5 s, including a 1-s pre-stimulus period and a 3.5 s stimulus presentation period, followed by a no-stimulus interval of at least 8 s before the next pre-stimulus period. We did not perform retinotopic mapping experiments, but imaging locations correspond approximately to an eccentricity of 1–10° from the fovea (Gattass et al., 1981, 1988; Fize et al., 2003; Polimeni et al., 2006). These imaging locations are thought to adequately avoid the S-cone-free zone, which is observed in the center of fovea and is approximately 0.35° in diameter (Curcio et al., 1991).

Image frames were analyzed offline using custom software written in MATLAB (Mathworks). For each stimulus presentation, the average of frames obtained during the pre-stimulus period (*R0*, 1.0–0 s before the stimulus onset) and the average of frames during the stimulus period (*R1*, 1.0–3.5 s after the stimulus onset) were calculated on a pixel-by-pixel basis, and then a map of reflectance change *ΔR/R* (response map) was generated as *(R1-R0)/R0*. To extract locally evoked reflectance changes (mapping signals: ∼0.5 mm) from large-scale non-stimulus-specific changes (global signals: several millimeters or more; Frostig et al., 1990; Tanigawa et al., 2016), each response map was convolved with a 1.0 mm × 1.0 mm median filter and subtracted from the original map (high-pass filtering). Using response maps, single-condition maps were generated by subtracting the blank condition from each stimulus condition, and subtraction maps were generated by calculating the difference between two stimulus conditions (Tanigawa et al., 2010). The single condition maps of eight hues were vector-summed in polar coordinate space on a pixel-by-pixel basis, where the eight hues were placed at 45° intervals in polar coordinates, and the pixel value for each hue was the vector magnitude. Color-coded angle maps of hue preference were then created based on the angles of the summed vectors (Bonhoeffer and Grinvald, 1996).

The border between V1 and V2 within the imaging region was determined based on an ocular dominance map (response to left eye minus right eye stimulations; Lu and Roe, 2008). In the imaging region, V2 was designated as the cortex between the lunate sulcus and the border between V1 and V2, and V4 as the cortex anterior to the lunate sulcus (Gattass et al., 1981, 1988; Fize et al., 2003).

### Decoding Analysis

For multivariate pattern analysis based decoding analysis, we trained a linear support vector machine classifier using the Liblinear Matlab toolbox (Fan et al., 2008) to predict which of the eight hues was presented based on the pattern of Δ*R/R* values in the high-pass filtered response maps as described above. To reduce the decoding analysis time, we applied binning to the response maps so that their 10 × 10 pixels (80 μm × 80 μm) became one pixel, creating a reduced map of 131 × 108 pixels. The values of the pixels in the ROI of this reduced map were used as features for the classifier. There was no noticeable difference in the classifier performance between the reduced map with 10 × 10 binning and those with a smaller reduction ratio (Supplementary Figure 1). The values of the features obtained for each stimulus presentation were *z*-normalized independently for each pixel using the mean and standard deviation (SD) for all presentations of that stimulus to avoid high feature values dominating the outcome of the classifier. To minimize the bias of the classifier, we sampled the same number of trials for each stimulus condition in the classification analysis. If the numbers of trials varied in respect of the conditions, we sampled randomly a subset of trials in the condition with more trials to ensure that all conditions had the same number of trials. We assessed decoding accuracy using cross-validation. In the 10-fold cross-validation procedure, the original dataset of features is randomly divided into 10 subsets. We then trained the classifier on nine of those subsets (the training dataset) and tested the accuracy of the classifier on one subset (the test dataset). This process was repeated 10 times, and the average accuracy was calculated. Classification accuracy can be seen as an indication of the amount of stimulus condition information available in the patterns of features (Gochin et al., 1994; Quiroga and Panzeri, 2009; Haynes, 2015).

### Statistical Analysis and Domain Size Estimation

To assess the statistical significance of the modulation between conditions in the response maps, we applied a two-tailed *t* test and ANOVA to the response map data from 40 presentations in each stimulus condition. *P*-values were calculated by these statistics in each pixel (statistical map). For multiple comparison correction, we adopted a cluster-extent based thresholding procedure (Woo et al., 2014) in which only regions that consisted of at least 200 contiguous pixels with *P* < 0.05, contained a pixel with *P* < 0.001, and reproduced in another session were regarded as domains with significant modulation; domains that did not meet these criteria were excluded. To remove high-spatial-frequency noise from the statistical maps, we smoothed each Δ*R/R* map before analysis using a 160 μm × 160 μm median filter. Signals from pixels on and near large vessels were less reliable because of large trial-by-trial fluctuation that occurred even without visual stimulation. To exclude these regions from the analysis, we calculated pixel-wise SD of blank-condition images across trials. Pixels with large SD (>the upper limit of 95% one-sided confidence interval based on the χ*^2^* distribution) were eliminated from further analysis (shaded in dark green in the statistical maps).

After the above processing, a region consisting of continuous significant pixels were considered to be one domain that responded selectively in each stimulus condition. The area of the domain was measured using ImageJ (National Institutes of Health, Bethesda, MD, United States). Domains containing pixels with large SD of response around large vessels estimated as described above were excluded from the area calculation. It should be noted that domain area in our measurement may depend on the method we employed, such as the size of the spatial filtering and the number of stimulus presentations. Therefore, the data of calculated domain areas were used primarily for relative comparisons between visual cortices and between hues.

We tested the statistical significance of the classifier’s performance using the bootstrap method. From the training dataset, we constructed a new dataset by randomly resampling the same number of trials as that dataset with replacement, trained a classifier on that dataset, and tested the performance of the classifier on an intact test dataset as described above. This procedure was repeated 1,000 times to create a bootstrap distribution of decoding accuracy, and its mean and 95% confidence interval were estimated. The proportion of values smaller than the chance level in that distribution was calculated as the *P*-value for the null hypothesis that decoding accuracy does not differ from the chance level. When examining the statistical significance of differences in classification accuracy, two bootstrap-sampled distributions were deemed statistically different if 97.5% of their bootstrap distributions were non-overlapping. If the observed *P*-value was lower than the lowest possible *p*-value achievable with our bootstrap method (*P* = 10^–3^ for 1,000 samples), it was set to *P* = 10^–3^. We set our threshold for significance at *P* < 0.05.

### Histological Procedures

After finishing all optical imaging sessions, for histology, monkey G was given an overdose of sodium pentobarbital (>50 mg/kg body weight, i.v.) and was perfused through the heart with sucrose solution [8.5% sucrose, 5 mM MgCl_2_ in 20 mM phosphate buffer (PB), pH 7.5], followed by 4.0% paraformaldehyde in 0.1 M PB. The brain was removed from the skull, and the visual cortex of the right hemisphere was separated from the rest of the brain. The brain block was immersed in PB with 30% sucrose at 4°C overnight until it sank into the bottom of the container. The imaged area of the visual cortex was cut tangentially at 40 μm using a freezing microtome. Sections were stored at −20°C in cryoprotectant solution [30% ethylene glycol, 30% glycerol, and 40% phosphate-buffered saline (PBS), pH 7.5] until used. Cytochrome oxidase (CO) histochemistry was conducted according to previous studies (Wong-Riley, 1979). Briefly, free-floating sections were washed in 5% sucrose in PBS a few times, then reacted with CO reaction solution which contained 200 μg/mL cytochrome C (Sigma-Aldrich, St. Louis, MO, United States), 150 μg/mL catalase (Sigma-Aldrich, St. Louis, MO, United States), and 100 μg/mL 3,3′-diaminobenzidine (DAB; Sigma-Aldrich) in 5% sucrose/PBS on a shaker (30 rotations per minute) in an incubator (37°C) for 12–24 h. Sections were then transferred into PBS buffer, washed for a few minutes, mounted on glass microscope slides, and air-dried. Sections were then dehydrated through increasing concentrations of ethanol and coverslipped with xylene-based glue. Sections with histochemistry were scanned with a VS-120 automated brightfield microscope (Olympus, Tokyo, Japan). Scanned images were edited with photo editing software, Adobe Photoshop (cc 2018, Adobe, San Jose, CA, United States) to align different kinds of histochemistry sections and OI maps. Photoshop was used only to scale, rotate, crop, and adjust the contrast of the image; these edits were applied to the entire image with the exception of cropping. Monkey G was also used in a separate tracer study (Liu et al., 2021), and some of the data presented here are also used there for other purposes.

## Results

**FIGURE 1:**
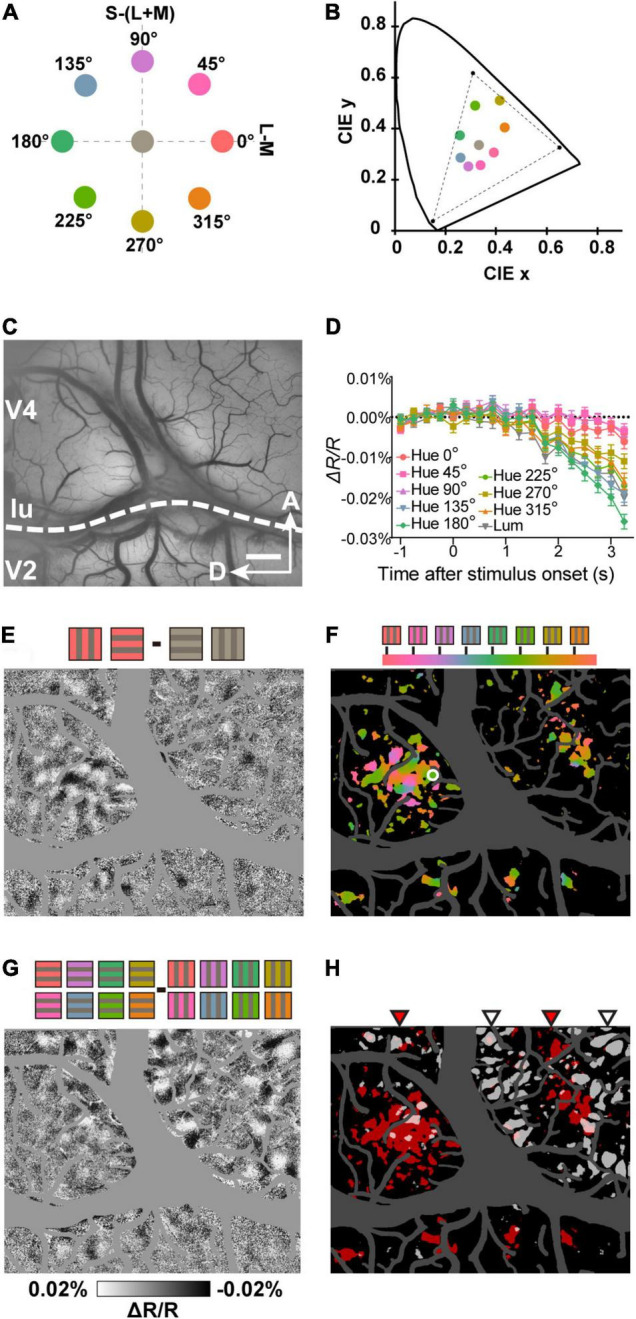
Imaging hue preference maps in visual cortex. **(A)** Location of the hues and gray used in the stimuli in the equiluminant plane of the DKL color space. **(B)** The same hues and gray in the CIE 1931 *xyY* chromaticity space. The dashed triangle indicates the gamut of the LCD monitor used for stimulation. **(C)** Example of an imaged region containing V2 and V4. **(D)** Time courses of average reflectance change at a sampled site (white circle in **F**) in response to the grating stimulus with each of the eight hues and one gray. Error bars represent standard error of the mean (SEM). **(E)** A subtraction map in response to Hue 0° minus achromatic grating stimuli (*n* = 160 presentations). Note that in the subtraction map, darker pixels indicate a stronger response for the first condition and lighter pixels for the second condition **(F)** A color-coded vector map of hue preference. Hue preference indicated by colors according to the key at the top. Colored regions represent pixels that had a strong selectivity for hues (one-way ANOVA, *n* = 640 presentations, *P* < 0.0001, uncorrected). **(G)** A subtraction map in response to horizontal minus vertical grating stimuli of eight hues. **(H)** Statistical maps created using two-way ANOVAs with hue (eight hues) and orientation (two orientations) as factors (*n* = 640 presentations, *P* < 0.0001, uncorrected). Red pixels represent regions with a significant main effect of stimulus hue (hue-sensitive regions) and Gray pixels represent regions with a significant main effect of stimulus orientation (orientation-sensitive regions). The red and gray triangles at the top indicate distinct clusters of hue-sensitive regions and orientation-sensitive regions in V4, respectively. lu, lunate sulcus, A, anterior, D, dorsal. Scale bar, 1 mm in **(C,E–H)**.

To study the functional organization of hue-selective response in the visual cortices, we conducted ISOI from the visual cortex (V1, V2, and V4) of four hemispheres of three anesthetized monkeys. Imaging in V1 and V4 was performed in all four hemispheres. Imaging in V2 was performed in only three hemispheres because one monkey did not contain an imageable V2 exposed in the chamber. We used color stimuli based on the DKL color space and selected hues at 0°, 45°, 90°, 135°, 180°, 225°, 270°, and 315°centered on the gray background hue (Figures 1A,B). The increasing and decreasing directions of the L-cone input in the L – M axis of the DKL color space (+L–M and +M–L) correspond to 0° and 180°, respectively, and the increasing and decreasing directions of the S-cone input in the S axis (+S and –S) correspond to 90° and 270°, respectively. Those four directions are called cardinal directions, and the other four directions are called intermediate directions. We will henceforth refer to these hues as the hue 0°, hue 45°,…, hue 315°, based on their polar angles in the DKL color space. Stimuli consisted of an equiluminant full-field gray presented for 1 s, followed by a square-wave grating (at 10% luminance contrast) comprising color/gray or achromatic dark gray/light gray with for 3.5 s. The grating was oriented horizontally or vertically. The color of the color/gray gratings was chosen from one of the eight hues described above.

In ISOI, due to the decrease in blood oxygenation associated with the neuronal response, the response areas exhibit a darkening of the tissue (decrease in reflectance; Frostig et al., 1990; Bonhoeffer and Grinvald, 1991). Some locations in the imaging region (an example shown in Figure 1C) exhibited differential reflectance change in response to each hue (Figure 1D). To obtain hue preference maps from the imaging region, we subtracted the response to the achromatic gratings from the response to the hue/gray gratings pixel by pixel. This allowed us to visualize the cortical areas that show a preferential response for that hue (Figure 1E; hue 0° minus gray). Color-coded vector maps of hue preference with statistical significance were also calculated (Figure 1F). Note the regularly spaced hue-sensitive areas in V2, which likely correspond to V2 color (thin) stripes (Lu and Roe, 2008). In the same region of V4, we also observed orientation-sensitive areas (Figure 1G). Consistent with previously described functional structures (Tanigawa et al., 2010), the color- and orientation-sensitive band-like areas were interleaved in V4 (Figure 1H).

### Hue Domains and Clusters in V1, V2, and V4

#### Hue Response Maps

**FIGURE 2:**
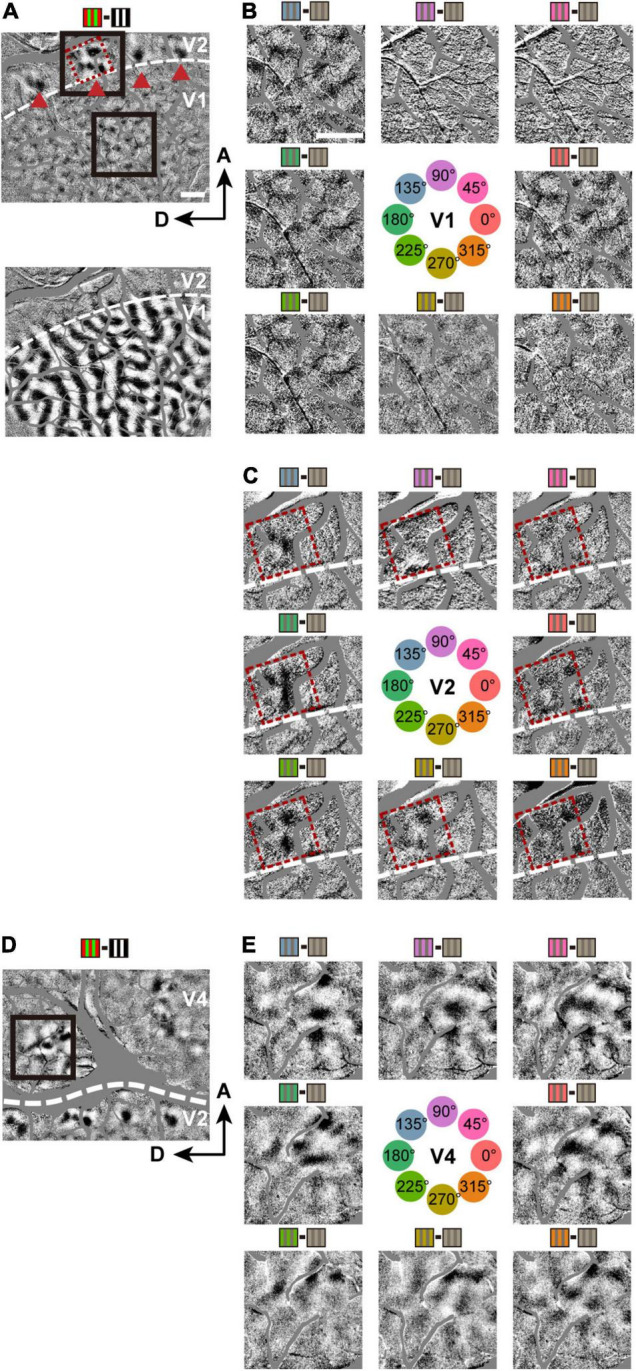
Hue maps in V1, V2, and V4. **(A)** Top: a subtraction map in response to isoluminant red/green minus luminance-contrast (100%) black/white grating stimuli (including V1 and V2 in Case 1), visualizing color-sensitive regions. Red dashed frames highlight a hue-sensitive region in V2. Triangles indicate the same four color-sensitive regions in V2 for both **(B,C)**; Bottom: A subtraction map in response to left eye minus right eye stimulations, determining the border between V1 and V2 (broken line). **(B)** Subtraction maps in response to individual hue minus achromatic grating stimuli, from the V1 region outlined by a lower black frame in the top panel of **(A)**. The hues used for the stimuli are indicated at the center. **(C)** Subtraction maps in response to individual hue minus achromatic grating stimuli, from a V2 region outlined by a upper black frame in the top panel of **(A)**. **(D)** A subtraction map in response to red/green minus black/white grating stimuli (including V2 and V4 in Case 1). **(E)** Same conventions as in **(B)**, but from a V4 region outlined by the black frame in **(D)**.

The above approach was used to study hue-selective responses in V1, V2, and V4 (Lu and Roe, 2008). First, a subtraction map was created from responses to red/green isoluminant gratings minus luminance-contrast black/white gratings (Figure 2A, top). In this map, regions that responded more strongly to red/green gratings are represented by darker pixels, while regions that responded more strongly to black/white gratings are represented by lighter pixels, and typical color-sensitive blobs in V1 and color-sensitive stripes in V2 that run roughly orthogonal to the V1/V2 border were found as in a previous study (Lu and Roe, 2008). The border between V1 and V2 was also revealed by a subtraction map in response to left eye minus right eye stimulations (Figure 2A, bottom). We then generated response maps of eight hues in the DKL color space at sites containing those color-sensitive regions in V1 and V2. In V1, a qualitative comparison of these maps shows robust response (dark areas) to the hues 0°, 135°, 180, and 225°, and relatively weaker response to hues 45° and 90° (Figure 2B). In V2, the response to the hue 90° hue remained relatively weak (Figure 2C). In V4, a subtraction map of red/green gratings minus black/white gratings revealed a relatively large population of color-sensitive regions (Figure 2D). The responses to all eight hues appeared more robust and more comparable in magnitude in V4 (Figure 2E).

#### Size of Hue Domains and Clusters

**FIGURE 3:**
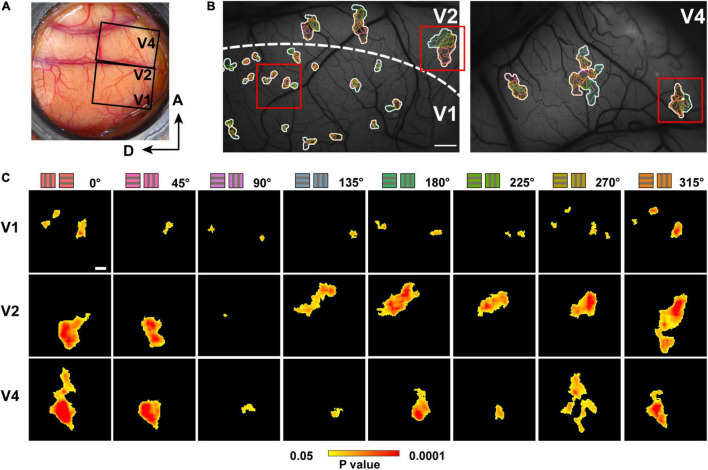
Hue domains and hue clusters in V1, V2, and V4. **(A)** A view of the exposed cortex over V1, V2 and V4 (Case 2). **(B)** hue domains (color contours) and hue clusters (white contours) in V1, V2, and V4 are displayed from the black framed region in **(A)**. Scale bar: 1 mm. **(C)** Examples of hue domains that responded significantly more strongly to stimuli of each hue (indicated on the left) compared to achromatic stimuli in V1 (left), V2 (middle), and V4 (right; pixel-wise two-tailed *t* test, *n* = 160, *P* < 0.05 and the peak *P* < 0.001, uncorrected). These were obtained from the regions framed in red in the respective visual areas in **(B)**. Scale bar: 100 μm. The color bar at the bottom shows a scale of *P* values on a log scale.

**FIGURE 4:**
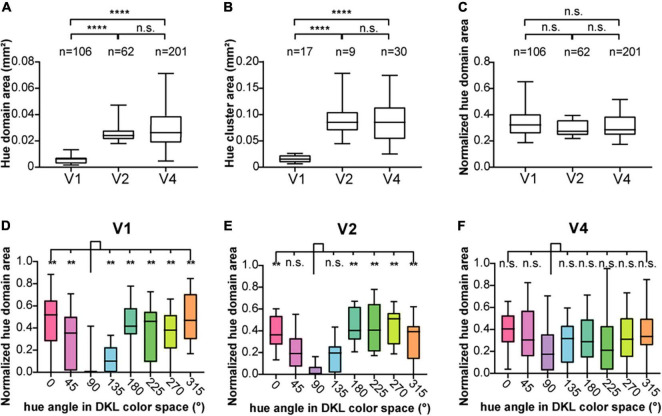
The size of hue domains and hue clusters in V1, V2, and V4. **(A)** Boxplot of the areas of hue domains in the visual cortices (*n* = 106, 62, and 201 hue domains for V1, V2, and V4, respectively) and their comparisons. **(B)** Boxplot of the areas of hue clusters in the visual cortices (*n* = 17, 9, and 30 hue clusters for V1, V2, and V4, respectively) and their comparisons. **(C)** Boxplot of the normalized hue domain areas (the area of each hue domain divided by the area of the hue cluster to which the domain belongs) in the visual cortices and their comparisons (the statistics in **(A–C)**, two-tailed *t* test, ****P < 0.0001; n.s., *P* > 0.05, Bonferroni corrected). **(D–F)** Boxplots of the normalized hue domain areas for individual hues in V1, V2, and V4 and their comparisons between hue 90° and other hues (two-tailed *t* test: **P < 0.01, n.s. *P* > 0.05, Bonferroni corrected).

In each of the cortices, we observed patchy dark areas in the hue response maps (Figure 2). We therefore defined a hue domain as a patchy area that showed a significantly stronger response to hue gratings than to achromatic gratings (Figure 3). Comparing the size of the hue domains in V1, V2, and V4, we found that the hue domains in V1 were the smallest, those in V2 and V4 were several times larger, and there was no difference in the domain sizes between V2 and V4 (Figure 4A; two-tailed *t* test, V1 vs. V2: *P* < 0.0001; V1 vs. V4: *P* < 0.0001; V2 vs. V4: *P* > 0.05). Assuming a circle with the same area as each domain, the average diameter was 84 ± 62 μm, 183 ± 106 μm, and 195 ± 144 μm (mean ± SD, *n* = 106, 62, and 201) in V1, V2, and V4, respectively.

We also found that domains responsive to close hues in the color space spatially overlap to form clusters (Figure 3B, hue clusters; Li et al., 2014). Those clusters tended to be distributed apart from each other, especially in V1 and V2. To quantify these clusters, we defined a hue cluster as a series of overlapping domains comprising at least four of the eight hues. As with the domain size, the clusters were smallest in V1, and those in V2 and V4 were several times larger. There was no difference in the size of the clusters between V2 and V4 (Figure 4B; two-tailed *t* test, V1 vs. V2: *P* < 0.001; V1 vs. V4: *P* < 0.001; V2 vs. V4: *P* > 0.05). We further compared the size of domains responsive to each hue, normalized by the size of the cluster, and found that the normalized patch areas were significantly different between the hues in V1 and V2, but not in V4 (Figure 4C; One-way ANOVA for the eight hues, V1: *P* < 0.0001, V2: *P* < 0.0001, V4: *P* > 0.05). Pairwise comparisons of the normalized domain area across hues showed that in V1, the normalized domain area for hue 90° was the smallest compared to those for the other hues (Figure 4D, two-tailed *t* test, *P* < 0.01); In V2, the normalized domain area for hue 90° was also smaller than those for all other hues except for hues 45° and 135° (Figure 4E, two-tailed *t* test, *P* < 0.01). On the other hand, in V4, there was no significant difference in the normalized domain area between hue 90° and the other hues (Figure 4F, two-tailed *t* test, *P* > 0.05). These results suggest that the relative size of neural populations responding to +S hue is smaller in the superficial layers of V1 and V2, but as the hierarchy of the visual cortex increases, the size of neural populations responding to hue becomes equal regardless of cardinal or intermediate hue.

#### Organization of Hue Clusters

**FIGURE 5:**
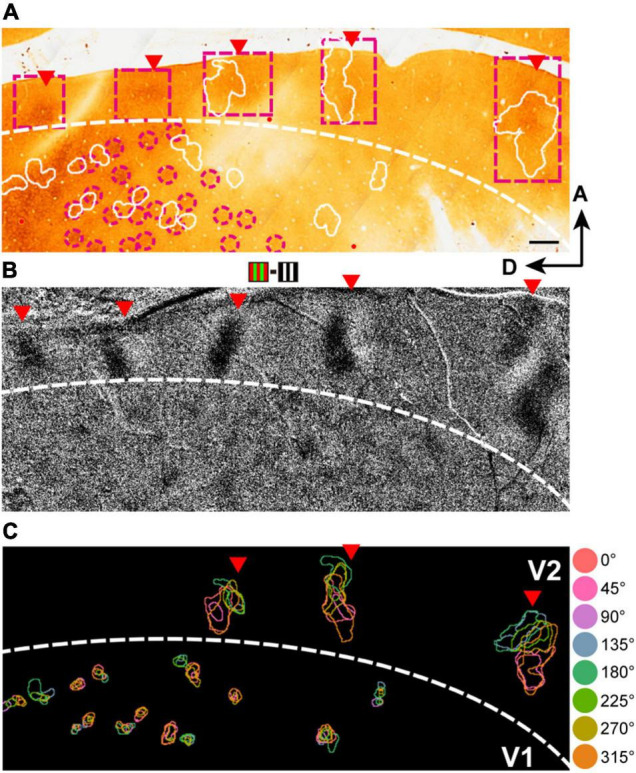
The organization of hue clusters in V1, V2, and V4. **(A)** A scanned image of a CO-stained tangential section from the imaged region in the V1 and V2 of case 2. The Section was cut mainly through the supragranular layer. The white contours indicate hue clusters in V1 and V2 in the imaged region (shown in **B**) that were aligned with the section with the aid of radially penetrating vessels. The magenta dashed frames indicate the locations of thin stripes in V2. The magenta dashed circles indicate the location of CO blobs close to hue clusters in V1. Scale bar, 500 μm. **(B)** A subtraction map in response to red/green minus black/white grating stimuli in the same cortical region as in **(A)**. Red triangles indicate patchy responsive regions in V2 in that map. For reference, the corresponding locations in **(A)** are also indicated by red triangles. **(C)** A map of hue domains in the same field of view as in **(B)**. The color contours indicate the locations of hue domains in individual hue clusters.

Here we illustrate an observation that the clusters containing domains of the eight hues were commonly found in V2 and V4, while the clusters in V1 often failed to contain all domains of the eight hues. Figure 5 shows an example of this observation. In this case, we obtained cytochrome-oxidase-stained tangential sections and aligned these histological sections with the optical images, based on the alignment of vessel holes in the sections with locations of radially penetrating vessels in the vessel map on imaging (Figures 5A,B; Roe and Ts’o, 1995; Kaskan et al., 2009). This alignment reveals correspondence of the locations of the dark cytochrome oxidase staining in V2 (Figure 5A, presumed thin stripes), differential responsive regions to red/green gratings vs. black/white gratings (Figure 5B), and hue clusters in V2 (Figure 5C).

**FIGURE 6:**
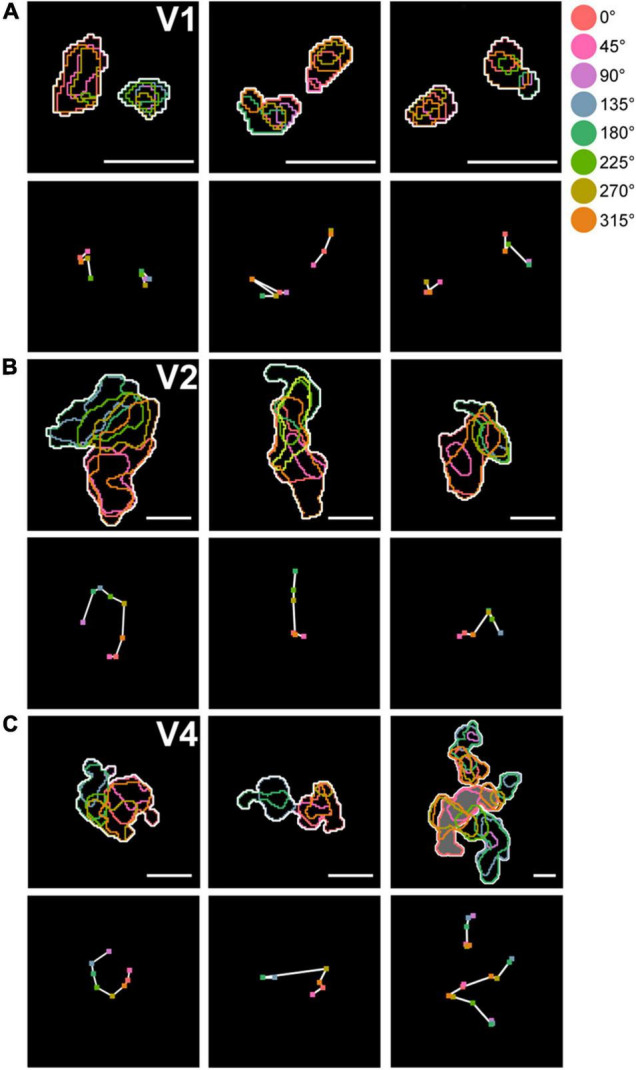
Arrangement of domains within hue clusters. **(A)** Top row: examples of arrangement of hue domains within hue clusters in V1. Color contours indicate hue domains, and white contours indicate hue clusters. Bottom row: Response peak points within individual hue domains are marked with colored dots. The peaks of adjacent domains in the hue angle are connected by white lines, showing their arrangement along the hue angle. **(B,C)** Same conventions as in **(A)**, but in V2 **(B)** and in V4 **(C)**. The upper right panel in **(C)** contains three clusters, with the uppermost cluster adjacent to another cluster. The lower two clusters merged and shared some hue domains (shown with gray background). Scale bar, 250 μm.

Defining a cluster containing all eight hue domains as a complete hue cluster, we found that incomplete hue clusters in V1 comprised 22.1% (30/136), while those in V2 and V4 comprised only 9.7% (7/72) and 12.5% (30/240), respectively. Domains of hue 90° tended to be most often missing. In addition, we found that within hue clusters in V2 and V4, hue domains were generally arranged linearly or in a circular manner in the order of hue angles in the color space (Figure 6). While in V1 and V2, hue clusters were distributed apart from each other, in V4, in addition to such separate clusters, some hue clusters were adjacent to each other or merged to share the same hue domains, indicating a more complex organization (Figure 6C, Right).

### Comparison of Hue Information in OI Maps Between Visual Cortices by Decoding Analysis

**FIGURE 7:**
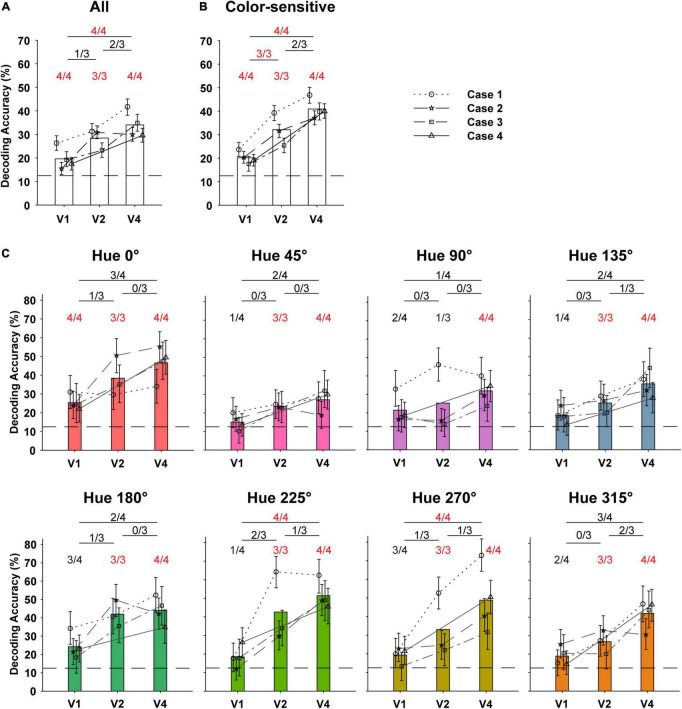
Decoding of hues in DKL color space using optical signals in the V1, V2, and V4. **(A)** Decoding accuracies of classifiers trained to identify the eight hues using patterns of optical signals obtained from different visual cortices (*n* = 4 for V1 and V4, *n* = 3 for V2) of four monkeys. For training the classifier, pixels of the response maps, each of which was binned to be 80.4 μm × 80.4 μm in size, were used as input features (see section “Materials and Methods”). In order to make comparisons between cortices, the number of input features (binned pixels) was kept the same across areas. We plotted the means of decoding accuracies obtained from 1,000 bootstrap procedures with error bars (95% confidence intervals). The bars show the average of the mean decoding accuracy for multiple monkeys, for visualization purposes. The number of cases in which the decoding accuracy was significantly higher than the chance level (12.5%) among the examined cases is shown above the bar (one-tailed bootstrap test, *P* < 0.05). The number of cortex pairs in which decoding accuracy was significantly different among the examined pairs was shown above the horizontal line at the top (two-sided bootstrap test, *P* < 0.05). If all cases or pairs were significant, the number is shown in red. **(B)** Decoding accuracies of hue classifiers trained using only signals in the color-sensitive regions identified by two-way ANOVA (see Figure 1H). **(C)** Decoding accuracies for each hue in the hue decoding using the signals in the color-sensitive regions.

If the activation patterns of the ISOI maps for two hues are different, it means that the patterns of the domain activity have information to discriminate the presentation of those two hues. Therefore, we examined the amount of information that could be decoded from OI maps of individual visual cortices (evaluated as the performance of a linear pattern classifier) about which of the eight hue stimuli was being displayed. The values of individual pixels of the OI maps per presentation, excluding the vascular regions, were used as input features for training and testing the classifier. In order to compare the amount of information across cortices, the number of pixels used was kept the same across cortices. The decoding accuracy of the classifier for the eight hues used was significantly higher than the chance level (12.5%) for all V1, V2, and V4 in all monkeys (Figure 7A; one-tailed bootstrap test, *P* < 0.05). When comparing V1 and V4, the decoding accuracy was higher in V4 for all monkeys (two-tailed bootstrap test, *P* < 0.05). Next, to prevent interference of signals from color-insensitive regions on decoding accuracy, we calculated decoding accuracy using only signals from regions that responded differently to the hues in each visual cortex (hue-sensitive regions, Figure 1H) as determined by pixel-wise two-way ANOVAs for the hues and orientations. In that calculation, the decoding accuracy was significantly higher in higher-order visual cortices between V1 and V2 as well as between V1 and V4 (Figure 7B). These results indicate that the patterns of population activity in V1, V2, and V4 had information that could discriminate the hues, and the patterns in the higher visual cortices tended to have more hue information.

Finally, we examined how well the classifier was able to classify individual hues (Figure 7C). The decoding accuracy for an individual hue indicates the degree to which the activity pattern by that hue was different from that by other hues. The results showed that in V1, only the decoding accuracy for the 0° hue was higher than the chance level for all monkeys (one-tailed bootstrap test, *P* < 0.05). This suggests that in V1, the activity patterns of the 0° hue were markedly different from those of the other hues. In addition, in V2 of all monkeys, decoding accuracy for all hues except the hue 90° was significantly higher than the chance level (one-tailed bootstrap test, *P* < 0.05). This may reflect the smaller size of the hue-90° preference domains in V1 and V2. In V4, decoding for all hues was significantly higher than chance, indicating that the activity patterns by individual hues were different from each other. Between visual cortices, decoding accuracies for hues 225° and 270° were significantly higher in V4 than in V1 for all monkeys, respectively, (two-tailed bootstrap test, *P* < 0.05). This suggests that the activation pattern by these hues became more distinctive for the other hues, especially from V1 to V4.

## Discussion

Using ISOI, we examined how activity at the neural population (domain) level in the superficial layers of visual cortices V1, V2, and V4 represent cardinal and intermediate hues in the DKL color space, which is based on cone opponency. We report three primary findings. First, in these visual cortices, we found hue preference regions (hue domains) that responded more strongly to specific hues in the DKL color space than to achromatic stimuli. Domains of different hues spatially overlapped to form clusters (hue clusters). In V1, consistent with previous studies (Xiao et al., 2007; Liu et al., 2020), hue preference domains were small in size and were located in blob-like clusters. In V2 and V4, they were larger and were organized in linear or circular arrangements according to the order of hue angle in the color space, forming separate clusters. In V4, in some cases, they were organized in more complex arrangements, with some clusters adjacent to or overlapping each other. Second, the balance of hue representation differed across visual cortices: in V1, the hue 90° (+S) preference domains were relatively small. In V2, similarly, the +S preference domains were relatively small. In contrast, V4 exhibited more comparable domain sizes for all hue preferences. Third, decoding of response patterns to the eight hues, including the cardinal and intermediate hues, showed an improvement in decoding accuracy as the V1-V2-V4 hierarchy increased. Thus, our data (hue domain size, hue cluster size, organization complexity, and decoding accuracy) suggest that, with increasing cortical hierarchy, hue domains become less focused on cone-opponency. Rather, individual hues are represented more distinctly, resulting in a more full representation of hues in color space.

### +S Preference Domains

Electrophysiological studies have reported that neurons in macaque V1 show a variety of hue selectivity, including cardinal and intermediate hues (Lennie et al., 1990; Hanazawa et al., 2000; Wachtler et al., 2003). The S-cone signals are thought to contribute significantly to the formation of hue selectivity in those neurons (De Valois et al., 2000). The S-cone signals from the koniocellular layers of the LGN target layer 4A and CO blobs of layer 2/3 in V1, while the L/M cone-opponent signals from the parvocellular layers terminate in layer 4C in V1. These signals work to form the hue domains in the supragranular layers of V1. A recent study using two-photon calcium imaging (Chatterjee et al., 2021) revealed that neurons with a preference for each cardinal hue, including the +S, clustered within the CO blobs, forming a micro-map of hues. Interestingly, when the authors used color stimuli with sinusoidal gratings, many hue-selective neurons were tuned to the +M–L or +L–M direction, even those that preferred the +S or –S direction when using uniform color stimuli (see their Supplementary Figures 6, 7). Their results seem to be consistent with our finding, based on square wave gratings as stimuli, of smaller +S hue preference domains. This suggests that the +S hue preference domains that respond to spatially structured color stimuli such as square-wave gratings are not yet developed in V1. It is possible that the +S hue preference domain could be visualized if ISOI were performed using spatially uniform color stimuli. In our results, the +S preference domains were more comparable in size to the other hue preference domains as cortical hierarchy increased. This could be interpreted as an enhancement of the +S signals from V1 in higher visual cortices, or it may simply be a result of the development of domains in those visual cortices that are able to represent a variety of hues.

### Effect of Macular Pigment on Domain Size Measurement

Macular pigment absorbs shorter wavelength light, and it covers the entire foveal region to block ultraviolet light to protect photoreceptors that are arranged at the highest density in the retina. Since the spectral absorbance of this pigment is higher in the shorter wavelength region, it is often misunderstood that this macular pigment reduces the intensity of blue rays alone: e.g., around 90° in hue angle in this study. One may argue that the reduced stimulation of bluish stimuli could have caused the reduced size of domains in V1 for hues around 90°. However, that is not the case. Due to the reduced energy of shorter wavelength light, the chromaticity of other colors also shifts toward an amber direction. Our numerical simulation of hue shifts by macular pigment density differences has revealed that the contrast of hue 90° stimulus with respect to the achromatic point was equal to that in hue 270° stimulus (Supplementary Figure 2 and Supplementary Table 1). Therefore, the relatively smaller domain size for hues around 90° observed in our V1 can’t be ascribed to any difference in absorption of shorter wavelength component by the macular pigment. It is rather plausible to consider the difference in the subcortical cone-opponent mechanisms.

### Decoding Suggests Increasing Hue-Based Representation

The decoding accuracy for the eight hues was lowest in V1 and increased for higher-order visual cortices. This may be partly because the hue domains were the smallest in V1. Smaller domains mean smaller neural populations preferring individual hues, which could result in a lower signal-to-noise ratio of the ISOI signal and/or individual ISOI signals reflecting multiple neural populations responsive to different hues. These factors may lower decoding accuracy. A human fMRI study showed that decoding accuracy for hues was highest in V1 compared to the rest of the visual cortex (Brouwer and Heeger, 2009). However, in that study, the authors used principal component scores as features, which were dimensionally reduced using principal component analysis from the available voxels in each visual cortex. The number of original voxels differed between the cortices because of differences in the physical sizes of the cortices, so caution is needed in interpreting their comparison of decoding accuracy across cortices. In another human fMRI study that examined decoding accuracy for intermediate hues across cortices using the same number of voxels with significant responses, the decoding accuracy in V2 was higher than that in V1 (Goddard et al., 2010), consistent with our results. In the decoding analysis results for each hue, the decoding accuracy for hue 0° was significantly higher in V1 than by chance in all cases in this study, suggesting that the pattern of neural population activity preferring the L-cone input is significantly different from the pattern of activity preferring other hues. This may reflect that the domains that prefer hue 0° in V1 are relatively large and possibly displaced from the other domains. Since hue 0° is a cardinal color, this is consistent with the human fMRI study showing that color representation in V1 is more dependent on cone opponent responses (Brouwer and Heeger, 2009).

### Possible Luminance Artifacts From the Hue Stimulus

Although the hue and achromatic stimuli we used to identify hue domains had the same photometric luminance, the actually perceived luminance may differ between individuals, and as such, unexpected luminance differences between stimuli (luminance artifacts) may have affected the results. Subtracting the response to the baseline stimulus (in our case, 10% luminance contrast achromatic stimulus) from the response to the color stimulus is an established method that has been used in previous studies of cortical color processing using ISOI (e.g., Tanigawa et al., 2010; Li et al., 2014; Liu et al., 2020) to avoid luminance artifacts. Therefore, we believe that in our experiments, we have taken maximum account to prevent possible luminance artifacts in stimuli with equal photometric luminance from affecting the results. Besides, since the maximum color modulation in L and M cone contrast in the hue stimuli we used was 7% at the maximum, the luminance artifact (if any) should be much smaller than that. Furthermore, the hue and achromatic stimuli each had 10% luminance contrast, and the hue domain was identified by the difference in responses to these stimuli. All color results were derived after limiting the response to color stimulus (photometric isoluminance) to those larger than the modulation in luminance stimulus (10% light gray/dark gray). Therefore, the effect of luminance artifacts, if any, should have been negligible. For S-cones, the magnitude was much larger in the cone contrast (70%) and might have carried luminance artifacts larger than 10%. However, if there were such systematic luminance artifacts in the S-cone stimuli, the decoding performance in V1 for +S hue stimuli should have been much better than +L–M or +M–L hue stimuli, but this was not the case. Therefore, we propose that even if there were luminance artifacts, the main results of the present study would not have been significantly affected.

### Toward a Functional Domain Basis of Perceptual Hue Representation

In sum, based on balance of hue spectrum representation, simple-to-complex clustering organization, and hue decoding accuracy, our findings suggest an increased emphasis on hue-based presentation with increasing cortical hierarchy. In particular, S cone representation relative to other hues becomes more prominent from V1, to V2, to V4, resulting in a relatively balanced representation of the hue spectrum in V4 (Tanigawa et al., 2010; Li et al., 2014). These findings reverberate with human fMRI studies which report the presence of cone-opponent and intermediate color directions in V1 (Parkes et al., 2009; Goddard et al., 2010; Kuriki et al., 2011), and responses in V4 which are more related to perceptual colors (Brouwer and Heeger, 2009). Together, the cellular, functional domain, and BOLD fMRI evidence support the view that each stage of the cortical hierarchy transforms color representation away from a cone-opponent representation and toward a perceptual hue-based representation (Brouwer and Heeger, 2009).

## Data Availability Statement

The raw data supporting the conclusions of this article will be made available by the authors, without undue reservation.

## Ethics Statement

The study involving animals was reviewed and approved by the Zhejiang University Institutional Animal Care and Use Committee. All procedures were performed in accordance with the National Institutes of Health Guidelines.

## Author Contributions

XD, AR, and HT designed the experiment. XD, XJ, TZ, and HT performed the experiments. XD, XJ, IK, and HT analyzed the data. TT performed histological analyses. XD, XJ, IK, AR, and HT discussed and wrote the manuscript. All authors contributed to the article and approved the submitted version.

## Conflict of Interest

The authors declare that the research was conducted in the absence of any commercial or financial relationships that could be construed as a potential conflict of interest.

## Publisher’s Note

All claims expressed in this article are solely those of the authors and do not necessarily represent those of their affiliated organizations, or those of the publisher, the editors and the reviewers. Any product that may be evaluated in this article, or claim that may be made by its manufacturer, is not guaranteed or endorsed by the publisher.
