# Supplementary material for: Representation of Cone-Opponent Color Space in Macaque Early Visual Cortices
Source: Front Neurosci. 2022 Jun 20;16:891247. doi: 10.3389/fnins.2022.891247 (PMC9251113; doi:10.3389/fnins.2022.891247)
Supplement: Supplementary file 1 [file Data_Sheet_1.DOCX]

Supplementary Material

## Supplementary Figures


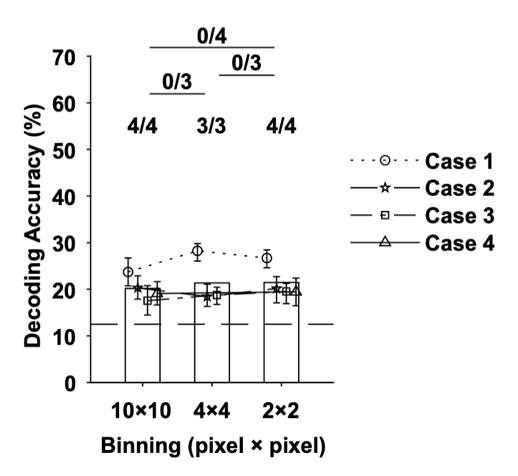


**Supplementary Figure 1. Effect of pixel binning on decoding accuracy in V1.** Decoding accuracy of the eight hues using the imaging signals in V1 for different pixel binning sizes. The horizontal axis indicates the number of binned pixels. For example, for 10 × 10 binning, the average value of 10 × 10 pixels was calculated and used as an input feature for a classifier. There was no significant difference in decoding accuracy in any hemispheric case between the 10 × 10 binning we used in this study and the 4 × 4 or 2 × 2 binning with smaller reduction rates.


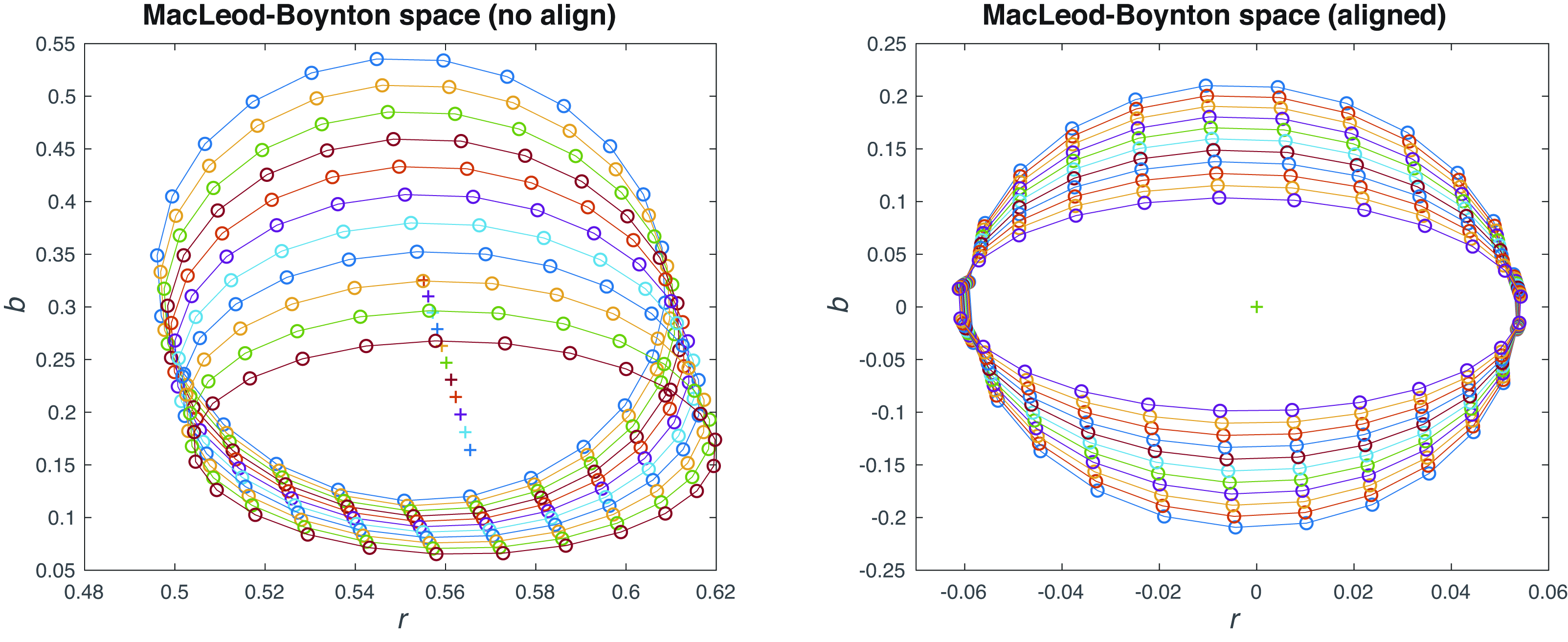


**Supplementary Figure 2. Effect of macular pigmentation on cone contrast of color stimuli.** The left panel shows colors along hue circle in cone-opponent space (MacLeod-Boynton space; MacLeod and Boynton, 1979), similar to the DKL space. The different ellipse shows chromaticity of color stimuli after the absorbance by macular pigment at various optical density. The most squashed ellipse in the bottom represents colors that passed through the densest macular pigment. Crosses at the center represent white point (equal energy white). Since color contrast must be assessed with respect to the achromatic point, the asymmetry between +S (90º in hue angle) and -S (270º) must be compared after aligned all elliptic loci to the corresponding achromatic point (crosses). The right panel show elliptic loci after the alignment, and it shows the extent along vertical axis (90º–270º direction) are symmetric with respect to the center cross.

| Color | x | y | Y(cd/cm^2^) |
| --- | --- | --- | --- |
| Hue 0º | 0.3811 | 0.3094 | 80 |
| Hue 45º | 0.3397 | 0.2700 | 80 |
| Hue 90º | 0.3012 | 0.2657 | 80 |
| Hue 135º | 0.2756 | 0.2962 | 80 |
| Hue 180º | 0.2776 | 0.3612 | 80 |
| Hue 225º | 0.3231 | 0.4355 | 80 |
| Hue 270º | 0.3875 | 0.4471 | 80 |
| Hue 315º | 0.4076 | 0.3811 | 80 |
|  |  |  |  |
| Red | 0.662 | 0.328 | 40 |
| Green | 0.320 | 0.613 | 40 |
| White | 0.319 | 0.318 | 80 |

**Supplementary Table 1.** **The CIE 1931-xy chromaticity coordinates of colors used in the visual stimulation.** The first column is the color in the visual stimulus, second and third column are the coordinate of the colors in CIE color space, the fourth column is the luminance of the colors.
